# Immunohistochemical Characterization of Connexin43 Expression in a Mouse Model of Diabetic Retinopathy and in Human Donor Retinas

**DOI:** 10.3390/ijms18122567

**Published:** 2017-11-29

**Authors:** Odunayo O. Mugisho, Colin R. Green, Jie Zhang, Nicolette Binz, Monica L. Acosta, Elizabeth Rakoczy, Ilva D. Rupenthal

**Affiliations:** 1 Buchanan Ocular Therapeutics Unit, Department of Ophthalmology, New Zealand National Eye Centre, University of Auckland, Auckland 1142, New Zealand; odunayo.rotimi@auckland.ac.nz; 2 Department of Ophthalmology, New Zealand National Eye Centre, University of Auckland, Auckland 1142, New Zealand; c.green@auckland.ac.nz (C.R.G.); jie.zhang@auckland.ac.nz (J.Z.); 3 School of Optometry and Vision Science, New Zealand National Eye Centre, University of Auckland, Auckland 1142, New Zealand; m.acosta@auckland.ac.nz; 4 Centre for Ophthalmology and Visual Science, University of Western Australia, Perth 6009, Western Australia, Australia; binz@iinet.net.au (N.B.); elizabeth.rakoczy@uwa.edu.au (E.R.)

**Keywords:** diabetic retinopathy, connexins, hyperglycemia, inflammation, Akimba

## Abstract

Diabetic retinopathy (DR) develops due to hyperglycemia and inflammation-induced vascular disruptions in the retina with connexin43 expression patterns in the disease still debated. Here, the effects of hyperglycemia and inflammation on connexin43 expression in vitro in a mouse model of DR and in human donor tissues were evaluated. Primary human retinal microvascular endothelial cells (hRMECs) were exposed to high glucose (HG; 25 mM) or pro-inflammatory cytokines IL-1β and TNF-α (10 ng/mL each) or both before assessing connexin43 expression. Additionally, connexin43, glial fibrillary acidic protein (GFAP), and plasmalemma vesicular associated protein (PLVAP) were labeled in wild-type (C57BL/6), Akita (diabetic), and Akimba (DR) mouse retinas. Finally, connexin43 and GFAP expression in donor retinas with confirmed DR was compared to age-matched controls. Co-application of HG and cytokines increased connexin43 expression in hRMECs in line with results seen in mice, with no significant difference in connexin43 or GFAP expression in Akita but higher expression in Akimba compared to wild-type mice. On PLVAP-positive vessels, connexin43 was higher in Akimba but unchanged in Akita compared to wild-type mice. Connexin43 expression appeared higher in donor retinas with confirmed DR compared to age-matched controls, similar to the distribution seen in Akimba mice and correlating with the in vitro results. Although connexin43 expression seems reduced in diabetes, hyperglycemia and inflammation present in the pathology of DR seem to increase connexin43 expression, suggesting a causal role of connexin43 channels in the disease progression.

## 1. Introduction

Diabetic retinopathy (DR) is a chronic disease that develops due to hyperglycemia-induced vascular and inflammatory disruptions in the retina with the vascular pathology characterized by blood–retinal barrier leakage, pericyte loss, endothelial cell death, and neovascularization [1]. Associated with these vascular changes is the stimulation of inflammatory pathways, such as secretion of pro-inflammatory molecules and immune cell activation, that worsen vascular pathology [2]. Together, these can cause diabetic macula edema characterized by fluid build-up in the macula and if untreated can lead to retinal detachment and vision loss [3].

While the vascular disorder in DR has been extensively studied, underlying molecular mechanisms remain enigmatic [4,5,6]. Much of the uncertainty around the molecular mechanisms governing the development and progression of DR stems from the differences in rodent models that are used to study the disease process [7,8]. Most in vitro and in vivo models described in the literature incorporate only hyperglycemia without accommodating for the role of inflammation in the disease process. The recently developed Akimba mouse, a genetic DR model, has provided a platform for more in-depth studies on the role of both hyperglycemia and inflammation in DR. It combines two previously existing animal models, the diabetic Akita mouse with a naturally occurring *Ins2* gene mutation, and the trVEGF029 Kimba mouse in which photoreceptors overexpress the human vascular endothelial growth factor (VEGF) protein [9,10]. Previous studies have shown that the Akimba mouse uniquely displays features of advanced DR, most of which are not present in other models of the diseases [9,10]. Wisniewska-Kruk et al. [10] showed that the Akimba mouse had reduced expression levels of various endothelial cell-specific markers such as von Willebrand factor and CD31 indicating a loss of blood vessels. Moreover, Rakoczy et al. [9] reported that the Akimba mouse showed a higher prevalence for extensive retinal edema that persisted with age compared to either of its parents. While the Akimba mouse has been described as a good model of advanced DR, it is important to note that the effect of VEGF overexpression precedes the manifestation of diabetic signs; hence, the model does not accurately mimic the sequence of disease development. Nonetheless, the Akimba model is the only mouse known to show signs of advanced DR, such as neovascularization and extensive macula edema [10].

Gap junctions are specialized, weakly selective communication channels that allow cells to directly transfer small molecules between each other. A complete gap junction is formed by two connexons or hemichannels, one from each of the two neighboring cells [11,12]. Each hemichannel is a hexamer that is formed from the oligomerization of six connexin subunits. Various connexin protein types are expressed throughout the body but the most commonly expressed in the retina is connexin43 [13]. The overexpression and opening of connexin43 hemichannels has been implicated in various diseases such as spinal cord injury [14,15], brain diseases [16], and retinal stroke [11,17,18,19]. Interestingly, these diseases are often characterized by vascular disruption and inflammation, the key pathologies in DR. In spinal cord injury, for instance, it has been shown that connexin43 hemichannels are overexpressed and this contributes to astrogliosis, microglial activation, and tissue damage [20]. Therefore, blocking of connexin43 channels has been shown to reduce tissue swelling and lesion spread to improve functional outcomes [14,21].

Interestingly, most studies investigating connexin43 in DR have suggested that its expression is decreased in the retina, which may be deleterious as it correlates with increased vascular cell apoptosis in both streptozotocin (STZ)-induced mice and rats [22,23]. Furthermore, in vitro studies have shown that hyperglycemia results in a decrease in connexin43 that leads to reduced gap junction activity and downregulation of tight junction proteins by endothelial cells, resulting in increased cell apoptosis [24,25]. A closer look at these studies revealed that hyperglycemia alone was used in the development of these in vitro and in vivo models. It is therefore possible that these are diabetes only models that do not necessarily portray the full range of pathology associated with DR. Therefore, it is important to distinguish between diabetic retinas and retinas with confirmed DR showing an element of inflammation to develop effective treatments for the different pathologies of non-proliferative DR (NPDR) and proliferative DR (PDR). An increase in connexin43 expression has also been observed in other CNS injuries or diseases associated with inflammation such as astrogliosis and microgliosis [14,17,26,27,28,29,30]. For instance, connexin43 expression was found to increase in a light damage model of age-related macular degeneration [31]. This is significant as several previous studies have reported no change in the retinal vasculature in diabetic models but extensive changes in the retinal vasculature in DR [9,10,32,33,34]. While sustained hyperglycemia is known to be a predetermining factor for DR, other vascular factors leading to inflammation also play a role in the development of the disease.

Therefore, the present study firstly evaluated the effect of hyperglycemia and inflammation, separately and in combination, on connexin43 expression in primary retinal microvascular endothelial cells (hRMECs) which line retinal blood vessels. It then determined changes in connexin43 expression in parallel with a marker of astrogliosis, glial fibrillary acidic protein (GFAP), in Akita and Akimba compared to wild-type mice, and correlated these to changes seen in retinas from human donors with confirmed DR diagnosis.

## 2. Results

### 2.1. High Glucose Exacerbates Pro-Inflammatory Cytokine-Mediated Increase in Connexin43 Expression

**Figure 1:**
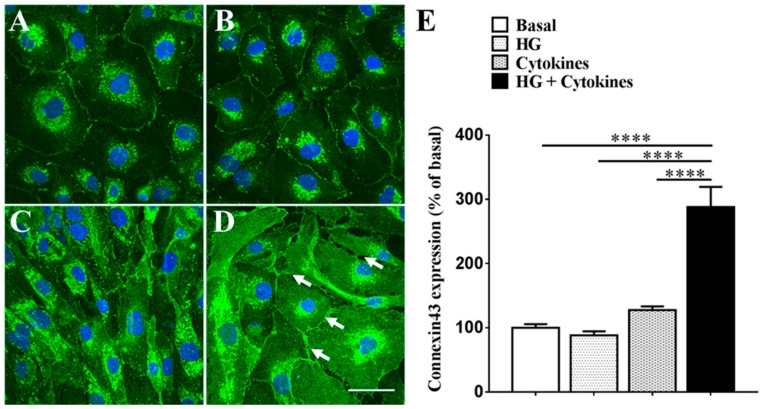
Effect of HG (high glucose) and pro-inflammatory cytokines on connexin43 expression in hRMECs. Immunohistochemical data showing connexin43 expression in (**A**) normal medium; (**B**) HG (25 mM); (**C**) pro-inflammatory cytokines (IL-1β and TNF-α 10 ng/mL each); and (**D**) a combination of HG and pro-inflammatory cytokines inducing a change in cell morphology with signs of cell swelling, possibly owing to hemichannel opening (indicated by white arrows); (**E**) Quantification of connexin43 expression relative to basal levels. Neither HG (*p* = 0.9569) nor pro-inflammatory cytokines (*p* = 0.6490) significantly increased connexin43 expression. However, co-application of HG and cytokines increased connexin43 levels relative to basal, HG, and cytokines alone (*p* < 0.0001 for all). Scale bar = 5 nm. Data presented as mean + SEM. Statistical comparisons were carried out using one-way ANOVA with Tukey’s multiple comparisons test; *n* = 6; *t* = 24 h; **** *p* ≤ 0.0001.

Figure 1 shows that high glucose (HG) (88.34 ± 6.05%) did not change connexin43 levels relative to the control (100.00 ± 5.51%; *p* = 0.9569) (Figure 1A,B). Similarly, pro-inflammatory cytokines (127.50 ± 5.81%) did not significantly increased connexin43 expression relative to the control (*p* = 0.6490) (Figure 1A,C). On the contrary, a combination of HG and cytokines (288 ± 31.28%) significantly increased connexin43 levels relative to the control, HG alone, and cytokines alone (*p* < 0.0001 for all) (Figure 1A–E). Additionally, there was a change in cell morphology following combination treatment with HG and cytokines (white arrows, Figure 1D) with cells appearing edematous possibly owing to hemichannel opening. The combination of HG and cytokines also lead to cytoplasmic connexin43 expression, which differed from the cell membrane localization seen in basal conditions as well as treatment with HG or cytokines alone. 

### 2.2. Connexin43 and Glial Fibrillary Acidic Protein (GFAP) Expression Increase in Akimba Compared to Wild-Type and Akita Retinas

**Figure 2:**
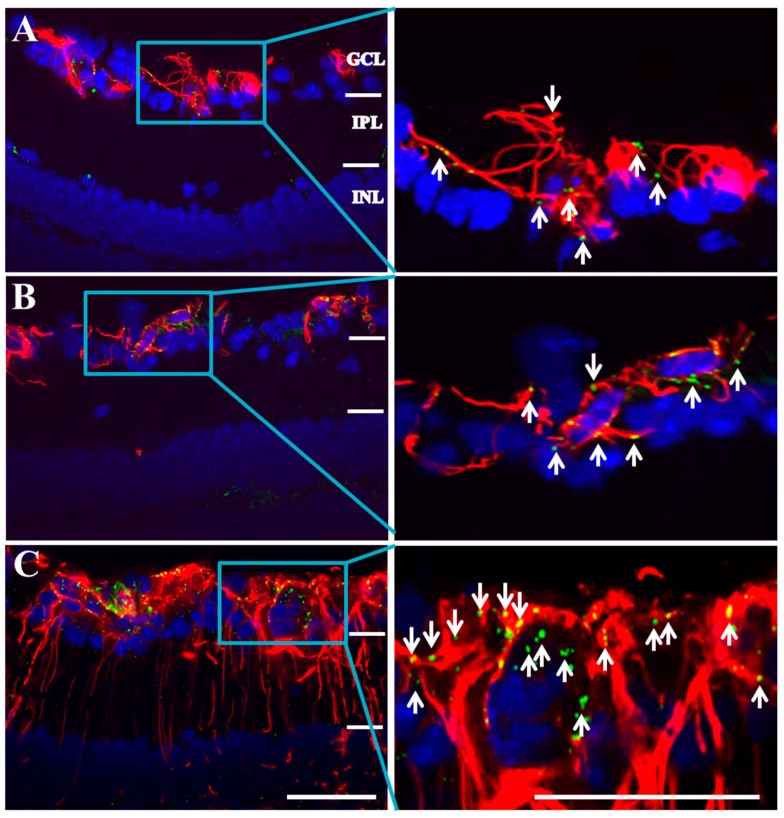
Connexin43 and glial fibrillary acidic protein (GFAP) expression in retinas of (**A**) wild-type; (**B**) Akita; and (**C**) Akimba mice. Connexin43 spots (green) were present in the GCL and co-localized with GFAP (red) in all mouse strains. GFAP labeling was evident within the GCL only in wild-type and Akita but spanned all retinal layers in Akimba mice. GFAP = glial fibrillary acidic protein; White arrows highlight connexin43 spots. GCL = ganglion cell layer; IPL = inner plexiform layer; INL = inner nuclear layer. Scale bar: 50 µm; *n* = 4 animals/group.

**Figure 3:**
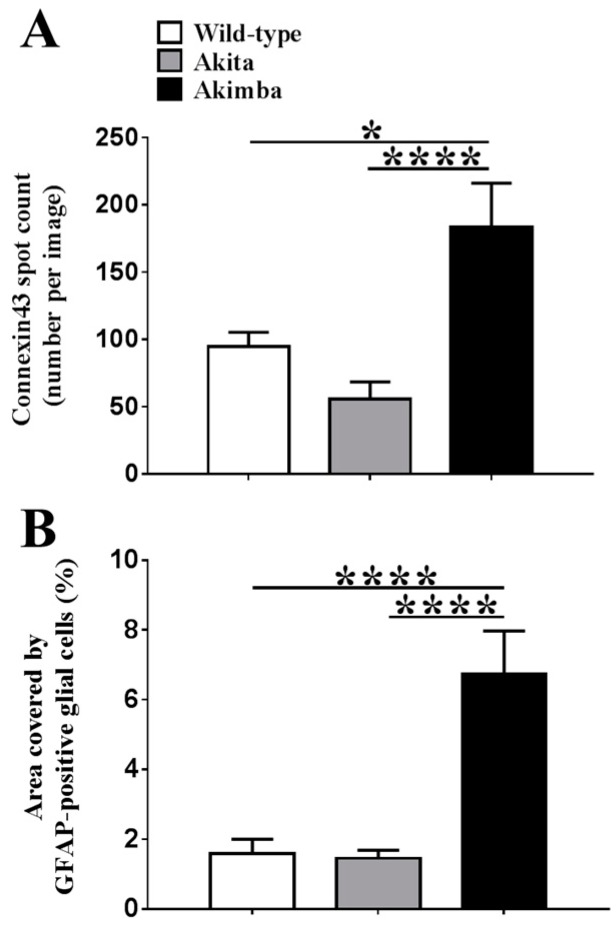
Quantification of (**A**) Connexin43 spots per image and (**B**) % area covered by GFAP labeling in wild-type, Akita, and Akimba retinas. There was no statistically significant difference in connexin43 spot counts between Akita and wild-type mice (*p* = 0.3047). However, the connexin43 spot count was higher in Akimba compared to wild-type (*p* = 0.0159) and Akita (*p* < 0.0001) mice. There was no statistically significant difference in GFAP labeling in Akita compared to wild-type mice (*p* = 0.9878). However, GFAP expression was significantly higher in Akimba compared to wild-type and Akita (*p* < 0.0001 for both) mice. Data presented as mean + SEM; statistical comparisons were carried out using one-way ANOVA with Tukey’s multiple comparisons test; *n* = 4 animals/group; * *p* ≤ 0.05, **** *p* ≤ 0.0001.

Figure 2 shows that connexin43 and GFAP co-expression was restricted to the ganglion cell layer (GCL) in wild-type and diabetic Akita retinas. Quantification of connexin43 spots (Figure 3) revealed that there was no statistically significant difference in connexin43 expression between wild-type (94.9 ± 10.5) and Akita retinas (55.6 ± 13.0; *p* = 0.3047). Similarly, there was no statistically significant difference in GFAP expression between wild-type (1.6 ± 0.4%) and Akita retinas (1.5 ± 0.2%; *p* = 0.9878). 

In Akimba retinas (183.3 ± 32.8), the connexin43 spot count was about two-fold higher compared to wild-type (94.9 ± 10.5; *p* = 0.0159) and Akita mice (55.6 ± 13.0; *p* < 0.0001). There was a four-fold increase in GFAP expression in Akimba (6.7 ± 1.2%) compared to wild-type retinas (1.6 ± 0.4%; *p* < 0.0001) and a five-fold increase compared to Akita retinas (1.5 ± 0.2%; *p* < 0.0001). Connexin43 expression appeared to be predominantly restricted to the GCL in Akimba retinas, while GFAP labeling was extended to Müller cells. This labeling pattern was indicative of astrogliosis and may also indicate Müller cell activation. Additionally, connexin43 spots were not always co-localized to GFAP in some areas of Akimba retinas, unlike wild-type and Akita eyes. For this reason, connexin43 expression, specifically in the blood vessel endothelium, was further investigated.

### 2.3. Connexin43 Expression Increases in Blood Vessels of Akimba Compared to Wild-Type and Akita Retinas

**Figure 4:**
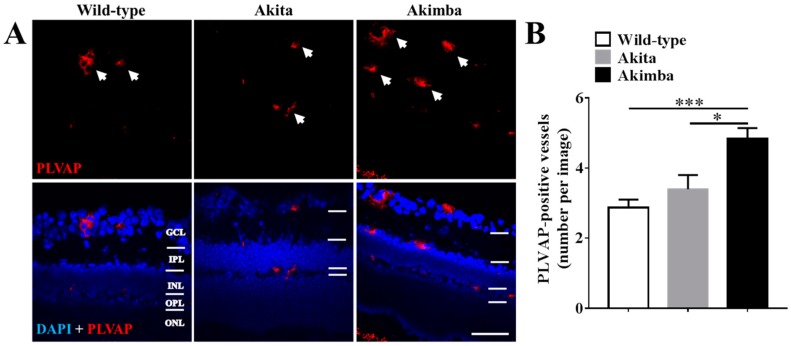
PLVAP-positive vessels in wild-type, Akita, and Akimba mice. (**A**) PLVAP (red) expression within retinal layers in wild-type, Akita, and Akimba mice. PLVAP-positive vessels are indicated by white arrows. GCL = ganglion cell layer; IPL = inner plexiform layer; INL = inner nuclear layer; OPL = outer plexiform layer; ONL = outer nuclear layer. Scale bar: 50 µm; (**B**) Quantification of PLVAP-positive vessels shows that there was no difference in vessel number between wild-type and Akita retinas (*p* = 0.4511). However, there were more PLVAP-positive vessels within the retina of Akimba mice compared to wild-type (*p* = 0.0005) and Akita (*p* = 0.0154) mice. Data presented as mean + SEM. Statistical comparisons were carried out using one-way ANOVA with Tukey’s multiple comparisons test; *n* = 4 animals/group; * *p* ≤ 0.05, *** *p* ≤ 0.0001.

As DR is primarily a chronic inflammatory vascular disease, it was important to evaluate changes in connexin43 expression within blood vessels specifically. PLVAP has previously been described as an endothelial cell-specific marker of newly formed blood vessels in mice [10]. However, before assessing connexin43 expression in blood vessels, the number of PLVAP-positive cells were compared between the three mouse strains to determine the extent of neovascularization (Figure 4). Results showed that there was no difference in PLVAP-positive vessels between retinas from Akita (3.4 ± 0.9) and wild-type (2.9 ± 0.6; *p* = 0.4511) mice. However, Akimba (4.8 ± 0.8) retinas had more PLVAP-positive vessels compared to wild-type (*p* = 0.0005) and Akita (*p* = 0.0154) retinas.

**Figure 5:**
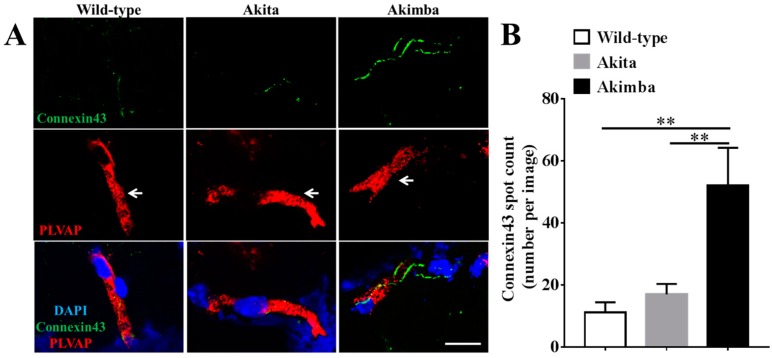
Connexin43 expression in PLVAP-positive blood vessels in the IPL of wild-type, Akita, and Akimba retinas. (**A**) Connexin43 expression (green) in blood vessels (PLVAP, red) in the IPL of wild-type, Akita, and Akimba retinas. Blood vessels are indicated by white arrows. Scale bar: 10 µm; (**B**) quantification of the number of connexin43 spots per blood vessel in wild-type, Akita, and Akimba mice. There was no significant difference in the number of connexin43 spots in blood vessels of Akita compared to wild-type mice (*p* > 0.9999). Akimba mice, however, showed a significantly higher connexin43 spot count compared to wild-type and Akita retinas (*p* = 0.0011 for both). Data presented as mean + SEM. Statistical comparisons were carried out using one-way ANOVA with Tukey’s multiple comparisons test; *n* = 4 animals/group; ** *p* ≤ 0.01.

To evaluate connexin43 expression changes in blood vessel endothelial cells in vivo, connexin43 co-localization with PLVAP labeling was assessed. Low connexin43 levels were observed on PLVAP-positive blood vessels of wild-type retinas (Figure 5A). PLVAP-positive vessels of diabetic Akita retinas (17.0 ± 3.3) showed a slight increase in connexin43 expression compared to wild-type retinas (11.2 ± 3.3), but this was not statistically significant (Figure 5B). Akimba retinas, on the other hand, showed significantly higher levels of connexin43 expression in PLVAP-positive vessels (52.8 ± 12.0) compared to wild-type (*p* = 0.0011) and Akita (*p* = 0.0011) retinas.

### 2.4. Connexin43 and GFAP Expression Increases in Human Donor DR Retinas Compared to Age-Matched Controls

**Figure 6:**
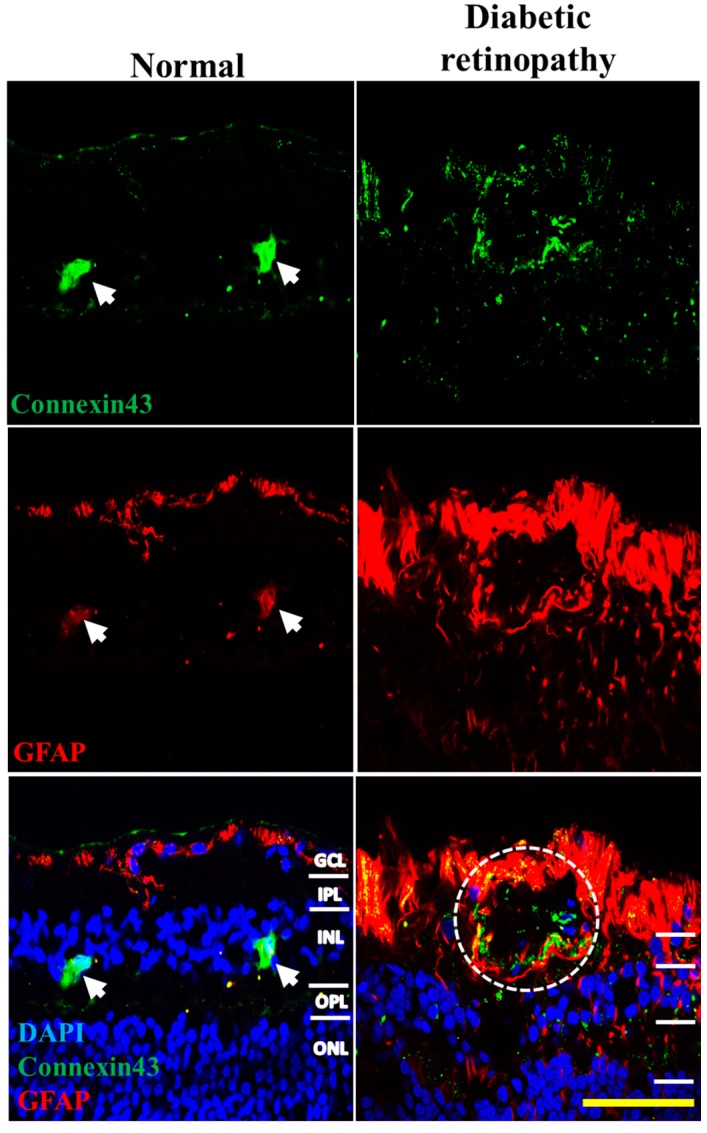
Connexin43 (green) and GFAP (red) expression in normal and human DR donor retinas in regions of extensive vascular damage. Large cells (white arrows, left column) represent non-specific auto-fluorescent amacrine cells. Connexin43 expression was markedly higher in the GCL of DR donor tissues compared to age-matched controls, and was strongly expressed throughout all retinal layers. GFAP labeling was also markedly higher in DR compared to normal donor eyes representing hyper-reactive Müller cells. Connexin43 expression was increased in regions identified as blood vessels and correlated with increased GFAP labeling at these sites, indicating glial cell activation (white circle). GCL = ganglion cell layer; IPL = inner plexiform layer; INL = inner nuclear layer; OPL = outer plexiform layer; ONL = outer nuclear layer. Scale bar: 200 µm.

Since Akimba is supposed to be a model of DR, as opposed to Akita, which is a model for diabetes or hyperglycemia only, we compared changes in connexin43 and GFAP expression seen in Akimba retinas with human DR donor retinas. Connexin43 expression was markedly higher in DR diagnosed donor retinas compared to age-matched controls (Figure 6). As with wild-type mice, connexin43 expression in normal human donor tissue was primarily co-localized with GFAP labeling of astrocytes in the GCL. However, DR donor retinas showed markedly higher connexin43 expression co-localized with GFAP particularly around blood vessels (white circle; Figure 6). Moreover, GFAP labeling in DR donor retinas spanned all retinal layers indicating activation of Müller cells. 

## 3. Discussion

It is increasingly accepted that the pathology of DR involves an interaction of hyperglycemic and inflammatory disease mechanisms. As a result, biochemical changes observed in DR may be different from diabetes (hyperglycemia) alone. Our results support this hypothesis as connexin43 expression patterns were quite different in HG-only conditions compared to a combination of HG with pro-inflammatory cytokines in vitro. Connexin43 expression increased with a combination of HG and pro-inflammatory cytokines compared to either treatment alone as well as basal levels. This suggests that hyperglycemia may increase the sensitivity of hRMECs to injury caused by inflammation such that the ability of the cells to cope with injury might become compromised. In this case, the increase in connexin43 triggered by cytokines alone was amplified by HG, possibly due to an HG-mediated increase in cellular metabolism and mitochondrial activity [35]. This is in line with a recent study reporting that HG alone did not induce changes in glucose transporter-1 (GLUT1) and pigment epithelium derived factor (PEDF) expression; however, a combination of HG and hypoxia increased the expression to a greater extent than hypoxia alone [36]. Furthermore, there was a change in cell morphology following combination treatment with HG and cytokines compared to either treatment alone. This could be a result of cell stress as it appears that cells are “pulling away” from each other. A change in cell morphology as a result of connexin43 hemichannel opening has been shown to cause cell edema eventually leading to cell death in cardiomyocytes [37]. Therefore, it is possible that the change in cell morphology is a result of connexin43 hemichannel opening.

Our animal data showed that, in diabetes-only Akita retinas, there was a decrease in connexin43 expression compared to wild-type retinas, though this was not statistically significant due to the high variability in expression amongst animals. This is in line with our in vitro results where HG alone did not induce any significant change in connexin43 expression. Previous in vitro and in vivo studies have suggested that hyperglycemia decreases connexin43 expression [25,38]. Bobbie et al. [22] demonstrated diabetes-induced downregulation of connexin43 expression using a diabetic mouse. Moreover, Tien et al. [39] found that connexin43 expression decreased in diabetic donor retinas compared to non-diabetic tissues. While these studies have described connexin43 expression in diabetes, our review of the literature found no studies that have directly assessed connexin43 expression in human retinas diagnosed specifically with PDR, the form of DR with the most extensive vascular disruptions. Our results showed that connexin43 expression increased significantly (*p* < 0.0001) in Akimba mice, a model of advanced PDR, and qualitatively in human donor tissues with confirmed PDR. 

It was proposed by Bobbie et al. [22] that a decrease in connexin43 expression in diabetes stimulates pathological mechanisms that result in DR with increased connexin43 expression being protective [25,38,40]. Our findings would indicate that, whilst connexin43 may well decrease with diabetes, it appears to be markedly increased with the onset of DR, associated with both astrogliosis and neovascularization. This suggests that an increase in connexin43 expression may be contributing to inflammatory mechanisms and vascular changes associated with the disease progression, as has been described for other retinal inflammatory conditions, both acute and chronic (for review, see [11]). Therefore, the difference in connexin43 expression observed between diabetic Akita and DR Akimba mice could highlight the concerted role of hyperglycemia and inflammation in DR. While the Akimba mouse exhibits both human VEGF over-expression and hyperglycemia, Akita mice show hyperglycemia only. Therefore, inflammation associated with over-expression of VEGF in Akimba mice seemed necessary to induce marked vascular breakdown seen in DR which was associated with an increase in connexin43 expression, similar to our hRMEC in vitro findings.

The extent of astrogliosis associated with increased connexin43 expression in Akimba mice was marked, while this was not seen in diabetic Akita mice. Additionally, PLVAP expression, a marker of newly formed blood vessels (in mice), was higher in Akimba retinas compared to those of wild-type and Akita mice. This is in line with previous studies where PLVAP expression was shown to be higher in Akimba compared to wild-type and Akita mice, suggesting the presence of neovascularization [9,10]. A recent study by McLenachan and colleagues [34], describing the properties of the retinal vasculature in normal, diabetic, and DR models, found no detrimental changes in vascular properties with diabetes but significant vascular breakdown in DR mouse models. This again suggests that DR and diabetes alone may have different vascular pathologies [34]. The same group previously reported that retinas of diabetic Akita mice showed no clinical signs of DR [32]. On the other hand, the Akimba mouse has been characterized as a model of advanced PDR [9,10]. These studies, in combination with our own findings, suggest that a marked increase in connexin43 expression may be associated with the pathology of DR. This pattern is not unique, as reduced connexin43 expression with diabetes, an overshoot in response to injury associated with skin wounds, has shown poor healing in both diabetic wound animal models and humans [41].

Connexin43 expression in Akimba retinas did not always co-localize with GFAP labeling, suggesting that connexin43-expressing endothelial cells may also show higher expression patterns. This was further supported by our in vitro studies where connexin43 expression was increased in primary endothelial cells. Therefore, we evaluated changes in connexin43 expression in blood vessels specifically and found that the connexin43 spot count was significantly increased in blood vessels of Akimba compared to wild-type and Akita mice. This increase in connexin43 may be detrimental as it may contribute to vascular leakage in vivo. A possible mechanism of connexin43-mediated vascular breakdown could therefore be an increase in connexin43 hemichannels on the endothelial cell surface compounded by pathological hemichannel opening within an inflammatory environment [11,27,42,43]. Given that an increase in connexin43 hemichannel expression and hemichannel opening has been associated with inflammation, apoptosis, and lesion spread in other vascular injury and disease conditions, such as spinal cord injury and retinal stroke, we would hypothesize a similar pathological pattern for DR [11,14,17,22,44]. 

In conclusion, our findings support the hypothesis that DR is characterized by a combination of both hyperglycemic and inflammatory disease mechanisms. Furthermore, they suggest that DR is associated with an increase in connexin43 expression that is distinct from the changes seen with diabetes or hyperglycemia alone. These findings therefore highlight the need for a careful consideration of models used to study the retinal pathophysiology of DR especially when evaluating novel treatments for the condition.

## 4. Materials and Methods

### 4.1. In Vitro Studies

Cell culture: Primary human retinal microvascular endothelial cells (hRMECs; Neuromics Antibodies, Minneapolis, MN, USA) were cultured in EGM-2 BulletKit medium (EGM-2; CC3162; Lonza, Basel, Switzerland) containing endothelial basal medium (EBM-2; CC3156; Lonza) and EGM-2 SingleQuots (CC4176; Lonza), supplemented with 10% fetal bovine serum (FBS; Invitrogen, Carlsbad, CA, USA). VEGF was excluded from the EGM-2 medium. Cells were grown in T75 flasks at 37 °C in a humidified 5% CO_2_ incubator and the medium was changed once every two days until confluent. 

High glucose and/or cytokine challenge: At passage 5–10, cells were plated at 2.5 × 10^5^ cells/mL in 8-well chamber slides before changing the culture medium to treatments in serum-free EBM-2 containing 1× antibiotic-antimycotic (15240; Thermo Fisher Scientific, Waltham, MA, USA). The glucose concentration in the EBM-2 medium was 5 mM. To study the effect of high glucose (HG) and pro-inflammatory cytokines on connexin43 levels, cultures were challenged with (1) no treatment (Basal); (2) 25 mM glucose (HG); (3) a combination of pro-inflammatory cytokines 10 ng/mL TNF-α (300-01A; Peprotech, Rocky Hill, NJ, USA) and 10 ng/mL IL-1β (200-01B; Peprotech) (Cytokines); or (4) a combination of 25 mM glucose, 10 ng/mL IL-1β, and 10 ng/mL TNF-α (HG + cytokines) for 24 h.

Immunohistochemistry of hRMECs: After 24 h of incubation in culture media containing treatments, hRMECs were fixed with 4% paraformaldehyde for 10 min and permeabilized with 0.1% Triton X-100 in phosphate buffer saline (PBS) for 10 min. Cells were then incubated with rabbit anti-connexin43 antibody (1:2000; C6219; Sigma-Aldrich, St. Louis, MO, USA) at 4 °C overnight followed by washing in PBS three times for 15 min. Goat anti-rabbit Alexa-488 (1:500; A11034; Life Technologies, Waltham, MA, USA) secondary antibody was applied to the cells and incubated at room temperature for 3 h. Goat anti-rabbit Alexa-488-only labeling was carried out as a control. Cell nuclei were stained with DAPI (1:1000; Sigma-Aldrich). Sections were washed and mounted using an anti-fade reagent (Citifluor™) and coverslips were sealed with nail polish. Experiments were repeated three times.

Image analysis and quantification of connexin43 plaques: All images were taken on an Olympus FV1000 confocal laser scanning microscope (Olympus, Center Valley, PA, USA) and processed using FV-10 ASW 3.0 Viewer and ImageJ software version 1.46r (National Institutes of Health). For connexin43 quantification, five images were taken per well, one in the center and the other four at clock positions 3, 6, 9, and 12 around the well. ImageJ was used to quantify the integrated density (area covered by connexin43 plaques × mean grey value), which was divided by the number of cells per image view with connexin43 expression presented as a percentage of the basal level. 

### 4.2. Animal Tissues

Preparation and immunohistochemical analysis of mouse tissues: All animal experiments were conducted in accordance with the Association for Research in Vision and Ophthalmology Statement for use of animals and with approval from the Animal Ethics Committee of the University of Western Australia (RA/3/100/1083, 1 April 2012). Twenty-four-week old male animals were used, as Akimba mice have previously been reported to develop signs of neovascularization at this age [10]. Sectioned eyes from wild-type C57BL/6, Akita (*Ins* −/−), and Akimba (*Ins* −/−, *VEGF +*/*−*) mice were kindly supplied by Dr. Binz, Lions Eye Institute in Perth, Australia [10]. Retinal sections were permeabilized in 100% chilled ethanol for 10 min and were then rinsed in PBS before blocking with 10% normal goat serum for 1 h at room temperature. Sections were subsequently incubated with the following primary antibodies overnight at 4 °C: anti-connexin43 (rabbit polyclonal connexin43; 1:2000; C6219; Sigma-Aldrich), anti-GFAP-Cy3 conjugated as a marker for astrocytes and hyper-reactive Müller cells (mouse monoclonal GFAP; 1:1000; C9205; Sigma-Aldrich) [45], and anti-PLVAP as a marker of blood vessel endothelial cells (mouse monoclonal MECA-32; Hybridoma Bank, Iowa City, IA, USA) [10]. Secondary antibodies used were goat anti-rabbit Alexa-488 (1:500; A11034; Life Technologies) or goat anti-mouse Cy3 (1:500; 115-165-003; Jackson Immuno Research, West Grove, PA, USA) and were incubated with sections at room temperature for 2 h. Nuclei were stained using DAPI (1 µg/mL; D9542; Sigma-Aldrich). Sections were washed and mounted using anti-fade reagent (Citifluor^TM^) and coverslips were sealed with nail polish. 

Image analysis and quantification of immunolabeling: All images were taken on an Olympus FV1000 confocal microscope and processed using FV-10 ASW 3.0 Viewer and Adobe Photoshop CS6 software. For connexin43 and GFAP immunohistochemical analysis, four animals were analyzed per group (*n* = 4) and six evenly spaced images were taken across each central retina giving a total of 24 images per group. This method ensured that representative images were taken for each retina and that similar locations were assessed between different eyes to avoid possible area bias between tissues. To measure connexin43 and GFAP levels within the retinal layers and connexin43 labeling per blood vessel, the drawing tool in ImageJ was used to select the desired areas. Each image was then split into its RGB channels with connexin43 in the green, GFAP/PLVAP in the red, and DAPI in the blue channel. The connexin43 spot count was carried out as previously described [45]. Briefly, the connexin43 image was converted to a binary image and an equal threshold value was applied to all images to reduce the background [46]. To separate different connexin43 clusters, a watershed binary algorithm was applied. The number of connexin43 spots was counted in the GCL where connexin43 expression was largely localized. 

For GFAP quantification, the GFAP image was converted to a binary image and an equal threshold value was applied to all images to reduce the background. The area (%) of GFAP was obtained by quantifying the area covered by GFAP labeling as a fraction of the total area spanning from the GCL to the inner nuclear layer (INL). ImageJ software was used to determine the % area covered by connexin43 or GFAP labeling relative to the total area selected. 

### 4.3. Human Donor Tissues

Preparation and immunohistochemical analysis of human tissues: donor eyes were collected by the New Zealand National Eye Bank in the Department of Ophthalmology at the University of Auckland, New Zealand, with approval from the Northern B Health and Disability Ethics Committee (NTX/06/19/CPD). The normal donor retina was obtained from a 67-year-old Caucasian female with no diagnosis of DR or any other ocular pathology with the retina excised 33.5 h after death. The DR donor retina was obtained from a 67-year-old Caucasian male with confirmed proliferative DR with the retina excised 8 h after death. Retinas were washed in PBS and passed through a sucrose gradient of 10%, 20%, and 30%, before embedding them in an optimal cutting temperature medium. For immunohistochemistry, sagittal 12 to 16 µm cryosections were mounted onto slides (Superfrost; Menzel-Gläser). Immunohistochemistry was conducted using the same protocol applied to mice tissues. PLVAP labeling, reported to be mouse-specific, was not carried out on human tissue samples. 

### 4.4. Statistics

Data are given as arithmetic means ± SEM. All statistical comparisons were performed using a one-way ANOVA with Tukey’s test to reduce any bias associated with multiple comparisons. A *p-*value < 0.05 was considered to indicate a statistically significant difference. All statistical analysis was performed in GraphPad Prism 6.
